# Mr. Atkinson, on Metallic Casts of the Ear

**Published:** 1803-10-01

**Authors:** James Atkinson

**Affiliations:** York


### *To the Editors of the Medical and Physical Journal.*

Gentlemen,

In the Tenth Volume of your Medical and Physical Journal, No. 54, I have observed a few remarks on the method of obtaining metallic casts from the osseous cavities of the human ear.

Your correspondent, under the signature of Remembrancer, expostulates with Mr. Low, on the originality of the invention of such casts. The result is, that he attributes the invention to Mr. Carlisle, surgeon, London.

Perhaps it may not be deemed intruding, to assert for myself, a supposed claim of precedency in preparing casts of the same parts.

In the year 1786, I advertised in York, a short course of anatomical lectures, &c. addressed to gentlemen of the profession, and to others of a philosophical turn. In that course, I shewed several preparations of the ear in wax; and in the lectures upon the organ of hearing, I demonstrated publickly the mechanism of the ear upon them.

Not having then prepared any in a more durable way, I recommended them to be prepared in some metallic form, or in plaster of Paris. But my recommendation I never fulfilled, although such was my intention.

The preparations then exhibited, I now have, some in a less perfect state than others, but sufficiently preserved to assert the fact.

I do not wish to detract in the least from Mr. Carlisle's claim to the invention; it is possible that two persons may have the same suggestions on like occasions.* There is notwithstanding, a certain degree of justice due to the industry

---

* John Hunter informs us, in his account of the organ of hearing in fishes, that he had prepared that organ by injection with wax and metals, prior to the year 1760. That great man, of course, is evidently entitled to the original idea, at least in respect to the present competitors.

industry of a man, from himself. This justice I assert, and no more.

I had certainly formed these injected preparations some years before they were publicly shewn.* But, as I am well aware of the indecency of petty contentions of this nature, and how much they have, in many instances, disparaged both science and civilization, I shall content myself with the liberty of only noting a fact, which many persons have witnessed, and (allow me to add) not one can disprove.

Never having seen any preparation of the sort in London, where I resided some years, nor having heard of any such, I did mention at the time, my method as a new and convenient one.

Whilst I was making a preparation of the ear, a sudden thought at once suggested to me the idea of injection.

My method was—to cover the temporal bone completely with clay, then to inject that bone; all foramina being previously stopped, which were judged necessary.

In this state the bone was corroded.

The injection failed in some instances in the semicircular canals.

*York,* I am, &c.

*Sept.* 14, 1803. JAMES ATKINSON.

* Either in the year 1782 or 1783, I know from circumstances.

